# The Evolution of Derived Monomorphism From Sexual Dimorphism: A Case Study on Salamanders

**DOI:** 10.1093/iob/obaa044

**Published:** 2020-12-21

**Authors:** Nancy L Staub

**Affiliations:** Department of Integrative Biology, Museum of Vertebrate Zoology, University of California at Berkeley, Berkeley, CA 94720, USA

## Abstract

While sexual dimorphism has long received special attention from biologists, derived monomorphism, the condition in which both males and females express similar derived features has been less well studied. Historically, the appearance of “male-like” features in females has been explained by the genetic correlation between the sexes. Recent work emphasizes the importance of studying the independent selective forces on both females and males to understand sexual dimorphism. Sexual dimorphism and derived monomorphism in the genus *Aneides* are examined in light of predictions of social selection. *Aneides hardii* shows the greatest degree of sexual dimorphism in snout–vent length and head width, with the other species of *Aneides* less sexually dimorphic. This reduced dimorphism, however, is not a return to an ancestral monomorphic state, but rather exemplifies derived monomorphism because females express traits that were limited in expression to males of ancestral species. Instead of calling these “male-typical” traits in females, I suggest the term “derived monomorphic” traits as these traits are typical in these females, and “derived monomorphic” can apply to both sexes. Increased attention to studying the patterns and ecological significance of derived monomorphism will shed light on the underlying selective forces, including sexual selection, on both females and males.

## Synopsis

**Spanish Resumen** El estudio del dimorfismo sexual ha recibido amplia atención en la biología. En cambio, el monomorfismo derivado, la condición en la que tanto los machos como las hembras expresan características derivadas similares, ha sido menos estudiado. Históricamente, la aparición de características “masculinas” en las hembras se ha explicado como el resultado de la correlación genética entre los sexos. Avances recientes han enfatizado la importancia de estudiar las fuerzas selectivas en machos y hembras independientemente. Éste trabajo analiza el dimorfismo sexual y el monomorfismo derivado en el género Aneides considerando predicciones de selección social. La especie Aneides hardii tiene el mayor grado de dimorfismo sexual en cuanto a la longitud hocico-cloaca y ancho de cabeza, mientras que las otras especies de Aneides son menos dimórficas. Sin embargo, este dimorfismo reducido no es una regresión a un estado monomórfico ancestral, sino un monomorfismo derivado puesto que las hembras expresan rasgos que se expresaban exclusivamente en los machos en especies ancestrales. Dado que estos rasgos son típicos en éstas hembras, propongo que en lugar de llamar a éstos rasgos “típicos masculinos,” se use el término de rasgos “monomórficos derivados”, que puede usarse para ambos sexos. El estudio de los patrones e importancia ecológica del monomorfismo derivado permitirá entender las fuerzas selectivas subyacentes, incluyendo la selección sexual, tanto en hembras como en machos. Translated to Spanish by Eleanor JS Weisblat and Laura Diaz-Martinez.

## Introduction

Sexual dimorphism, differences between males and females in secondary sexual characteristics, has long interested biologists in part because it is unexpected. Perhaps, because of this, sexual dimorphism has been widely studied (e.g., Ralls 1977; Slatkin 1984; Stamps 1993; Fairbairn 1997; Butler et al. 2007; Cox and Calsbeek 2009; Stewart and Rice 2018; Houle and Cheng 2020). Sexual dimorphism is not expected, or should be slow to evolve on theoretical grounds, because of the genetic correlation between sexes (Lande 1980), at least over short time scales (McGlothlin et al. 2019). While the genetic correlation between males and females can restrict the extent of dimorphism (Poissant et al. 2010), the historical focus on sexual selection on males contributed to the perspective that many female secondary sexual characteristics (traits developing at sexual maturity) that were similar to those in males, were merely a consequence of this genetic correlation and not a result of selection on the female in her own specific social context (Amundsen 2000). Researchers have argued that the evolution of the female phenotype can be better explained by independent selection on females (e.g., Ralls 1976; Burns 1998; Amundsen 2000; Clutton-Brock 2007, 2009). Furthermore, identifying the selective forces underlying sexual dimorphism illustrates that differential selection on both sexes determines the extent of sexual dimorphism (blackbirds: Irwin 1994; tanagers: Burns 1998; house finches: Badyaev and Hill 2000; *Drosophila:*  Chenoweth et al. 2008).

In comparison to sexual dimorphism, monomorphism has received less attention because it is predicted from simple theoretical expectations. Monomorphism is assumed to be the ancestral state for most groups and hence seems less interesting; there is no obvious signature of selection differentiating the sexes. Sexual monomorphism, however, can be a derived condition, evolving from sexual dimorphism. Derived monomorphism is the condition in which both males and females express the same derived features and thus are monomorphic, but differ from an ancestrally monomorphic condition as well as from the sexually dimorphic intermediate. Darwin (1871) was the first to identify this pattern of derived monomorphism and called it transference. He observed that females of some bird species possessed bright coloration and elaborate plumage and consequently closely resembled males, whereas in other species, females appeared drab and plain-colored. He proposed that from color dimorphism, color monomorphism was achieved through the transference of male characteristics to the female.

In regard to the differences between the females within the same genus, it appears to me almost certain, after looking through various large groups, that the chief agent has been the greater or less transference to the female of the characters acquired by the males… (Darwin 1871, 793).

West-Eberhard (2003) argued that derived monomorphism, or cross-sexual transfer as she called it, is an important cause of evolutionary novelty via social selection. I examine the patterns of sexual dimorphism and monomorphism in the salamander genus *Aneides* (family Plethodontidae).

Social selection occurs in the context of interactions among conspecifics and is defined as “differential reproductive success… due to differential success in social competition, whatever the resource at stake” (West-Eberhard 1983, 158). Social selection predicts sexual dimorphism if the involvement of females and males in relevant social interactions is unequal and predicts the evolution of derived monomorphism from dimorphism when both sexes are involved nearly equally (West-Eberhard 1983). Sexual selection then is a type of social selection that typically results in sexual dimorphism. Ecological causes of sexual dimorphism that involved social interactions at some point in their evolution, such as competitive displacement (Slatkin 1984; Hedrick and Temeles 1989; Shine 1989), are also included within social selection.

Derived monomorphism, and the social context of its evolution, has been studied in varied taxa. For example, social competition is argued to result in exaggerated yet monomorphic coloration in lek-breeding birds (Trail 1990) and hummingbirds (Bleiweiss 1985). In hyenas (*Crocuta crocuta*), females express androgenized traits compared to other carnivores, including external genitalia, increased body size and weight, and aggressive behavior (Matthews 1939; Dloniak et al. 2006; Hammond et al. 2012). Derived monomorphism has also been reported in ungulates (Kiltie 1985; Geist and Bayer 1988) and butterflies (Vane-Wright 1980, 1984; Clarke et al. 1985; Cook et al. 1994). Plants also show derived monomorphism (primroses: Mast et al. 2006). One of the earliest uses, perhaps the first, of the term derived monomorphism is in a description of derived monomorphic pollen (Baker 1948). This too was set in a social context of the evolution of self-incompatibility and a potential adaptation to sparse pollinators (Baker 1948).

Derived monomorphic females are often referred to as being “masculinized” or expressing “male-typical” traits. These terms are problematic, however, as these traits are typical female traits in these species with derived monomorphism. A more appropriate term for these traits is simply “derived monomorphic,” which refers to traits in one sex (male or female) that were ancestrally limited in expression to the other sex (male or female). One can imagine it would be useful to retain the information of which sex evolved the trait initially, in which case the longer term “male-to-female derived monomorphism” could be used, for example. Fanged frogs (*Limnonectes blythii*) of Southeast Asia show some female-to-male derived monomorphic traits such as low androgen levels, lack of nuptial pads, and parental care (Emerson et al. 1997).

Another potential case of derived monomorphism is if one sex loses its derived sexually dimorphic trait over time and the resulting monomorphic state is thus derived. I focus on the case, however, in which one sex evolves the expression of a derived trait that is ancestrally limited in expression in the other sex.

### Sexual dimorphism in plethodontids

Sexual size dimorphism is common in salamanders of the family Plethodontidae but typically is not profound, with females slightly larger than males (Shine 1979; Bruce 2000; Kupfer 2007). In amphibians, female body size is correlated with egg number, and thus female-biased size dimorphism is thought to primarily result from fecundity selection (Salthe and Duellman 1973; Shine 1979), although other factors may be important as well (Shine 1988). In a review of sexual size dimorphism among families of amphibians, selection on female body size was found to be the driver behind most sexual dimorphism (De Lisle and Rowe 2013). Important exceptions to this general pattern include the plethodontid genera *Phaeognathus* and *Desmognathus*, which have male-biased sexual dimorphism in body length (Bruce 1993, 2000; Bakkegard and Guyer 2004; Camp et al. 2019).

**Fig. 1:**
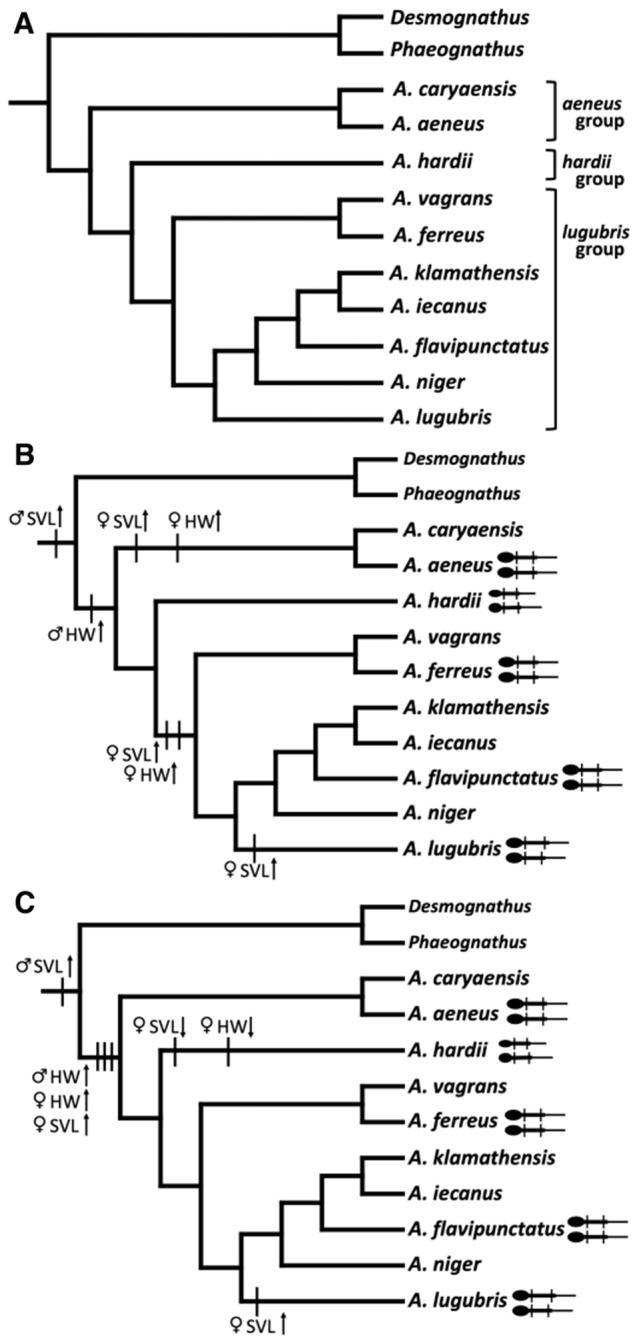
(**a**) Phylogeny of *Aneides*. There are 3 distinct species groups: the *lugubris* group, the *hardii* group, and the *aeneus* group (Wake, 1963). Tree is adapted from Vieites et al. (2007), Shen et al. (2016), Patton et al. (2019), Reilly and Wake (2019), and Jackman (1998); branch lengths are not representative of evolutionary change or time. (**B**) Phylogeny showing one hypothesis for the evolution of derived monomorphism in *Aneides.* Sexual dimorphism in body length, with males being longer than females, is ancestral for *Aneides.* HW dimorphism is a synapomorphy of *Aneides*, with male heads wider than females. Derived monomorphism has evolved in the *aeneus* and *lugubris* species groups, with females expressing derived monomorphic traits of jaw muscle hypertrophy and larger size. Female *A. lugubris* show a derived increase in SVL. (**C**) For this hypothesis of the evolution of derived monomorphism in *Aneides*, derived monomorphism is a synapomorphy of the genus (males and females do not differ in body length or HW) and subsequently female *A. hardii* lost the derived monomorphic traits in HW and SVL, and female *A. lugubris* increased in SVL. In the cartoon diagrams, the female is above the male in each pair.

Because *Desmognathus* and *Phaeognathus* are sister-taxa to *Aneides* (Fig. 1), but more distant relatives (e.g., *Plethodon, Karsenia*) are monomorphic, male-biased size dimorphism appears to be ancestral for *Aneides*. Analyses based on sequences of 50 nuclear markers find that *Aneides* diverged from a common ancestor with *Desmognathus* about 38–45 mya (Shen et al. 2016). The genus *Aneides* is characterized by (among other features) a single, rather than a paired, premaxillary bone (shared with the desmognathans among plethodontines) and by a rearrangement of the carpal and tarsal mesopodial elements (Wake 1963, 1966), the latter unique to *Aneides* among plethodontines. These 2 features, considered as key innovations, form the basis of a suite of morphological and ecological features associated with strengthening the skull and grasping ability (Wake 1963, 1966; Larson et al. 1981). In addition, the species of *Aneides* examined thus far exhibit dimorphism in the degree of jaw muscle hypertrophy and in features of the skull, including dentition (Wake 1963; Lynch 1981; Staub 2016).

Derived monomorphism may not be unusual, although it appears to be under-reported in the literature, in part because it requires knowledge of phylogenetic relationships. Because of the morphological variation both within and between species of *Aneides*, and because the phylogenetic relationships have been well-examined, this group provides a case-study for examining patterns of sexual dimorphism and derived monomorphism. If the social selection in both sexes has been important in the evolution of *Aneides*, I predict the evolution of derived monomorphism from the sexually dimorphic ancestral state in *Phaeognathus* and *Desmognathus*.

## Materials and methods

Head width (HW; maximum width of the head), snout–gular length (tip of snout to gular fold), snout–vent length (SVL; tip of snout to posterior margin of vent), axilla–groin (AG) or trunk length (distance between limbs), and tail length (posterior edge of vent to tail tip) were measured using 823 *A. hardii*, 452 *A. aeneus*, 358 *A. ferreus*, 426 *A. flavipunctatus*, and 363 *A. lugubris* specimens from the collections of the Museum of Vertebrate Zoology (MVZ), University of California at Berkeley, Field Museum of Natural History (FMNH), University of Michigan Museum of Zoology (UMMZ), the American Museum of Natural History (AMNH), and specimens from the personal collections of R. Highton, J. Beatty, and the author (NLS) (see Supplementary Appendix S1). Specimens were only used if they had been fixed and processed in the same way (fixed in 10% neutral buffered formalin, rinsed in water, stored in 70% ethanol) to avoid inconsistent distortion of morphology (Pierson et al. 2020). While head size and shape are complex variables, HW and HL were used as a simple index of a suite of morphological features of the salamander head. For adults, sex was determined by the presence (in males) or absence of mental glands, by the presence of ova in females, and by vent characteristics (smooth folds in females, papillose walls in males). Juveniles and adults without obvious mental glands or ova were sexed via dissection. Size at sexual maturity was determined by these dissections and was based on the literature (Lynch 1981; Wake et al. 1983; Waldron and Pauley 2007). Once the approximate size of sexual maturity was determined, the average SVL of at least 15 of the smallest sexually mature animals of each sex was taken and used for the specific size at sexual maturity for statistical analyses, acknowledging that size at sexual maturity varies across time and space and individual history. Sizes at sexual maturity were: *A. hardii*: male and female 45 mm SVL; *A. aeneus*: male 42 mm SVL, female 43 mm SVL; *A. ferreus:* male 51 mm SVL, female 53 mm SVL; *A lugubris:* male 51 mm SVL, female 55 mm SVL; *A. flavipunctatus:* male and female 54 mm SVL (Staub 2016). Data were transformed to their natural logarithms for analysis of covariance (ANCOVA), although for ease of viewing, graphs show untransformed measurements. Patterns of sexual dimorphism for SVL, HW, and HL have been described for *A. flavipunctatus* (Staub 2016); consequently, this species was only included for trunk length comparisons and for interspecific comparisons. Because of the variability due to tail regeneration, tails were not included in the analysis.

To test for SVL dimorphism, distributions of sizes were compared between adult males and females for each species using the Kolmogorov–Smirnov test (JMP version 14.2). Comparing the overall distribution of sizes avoids the problem of comparing different samples (sexes in this case) based on size, when the groups intentionally include animals of different sizes, if size at maturity differs between the sexes. To compare patterns of sexual dimorphism for other morphological variables, data were analyzed at 2 levels. First, males, females, and juveniles were analyzed intraspecifically using ANCOVA (JMP version 14.2; with lnSVL as covariate) to test for sexual dimorphism in HW, HL, and trunk length. Second, to compare traits relative to SVL between species, morphological variation was analyzed interspecifically. Because some dimorphism was present within species, males and females were analyzed separately across species using ANCOVA (JMP version 14.2) for each variable. A JMP add-in (One-way ANCOVA with Interaction Simple Slopes Test) was used to conduct unplanned pairwise comparisons of regression coefficients (using a Bonferroni correction producing a significance level of *P* ≤ 0.0025). When appropriate (when slopes were not different), intercepts among multiple species were compared using the Tukey Honest Significant Difference  (HSD) test (using a Bonferroni correction producing a significance level of *P* ≤ 0.0025).

For *A. hardii*, numbers of trunk vertebral elements were counted in cleared and stained specimens (Hanken and Wassersug 1981) and from X-rays. The chi-square test (Excel version 16.16.19) was used to compare trunk vertebral numbers between adult male (*n* = 90) and female (*n* = 134) *A. hardii*.

ANCOVA tests for differences among regression coefficients and intercepts of different groups. If the regression coefficients are not statistically different, the relationship between the 2 variables under study is similar. If this is the case, the next step in ANCOVA is to compare the means of the groups, adjusted for differences in the covariate; this step is commonly referred to as comparing the intercepts of the groups. If the intercepts (adjusted means) are significantly different, there are absolute differences among groups in the feature measured by the dependent axis (e.g., HW); the different groups are best represented by parallel lines with different intercepts. If the intercepts (adjusted means) are not different, one regression equation can describe all the groups being compared.

Differences in regression coefficients between groups indicate that the relationship between the variable under study (e.g., HW) and SVL (the covariate) differ and intercepts are not compared; if lines are not parallel, it is meaningless to compare their intercepts. For the following analyses, a larger regression coefficient indicates that HW, for example, is relatively larger at a given body length than it is for the group with a smaller regression coefficient. This means that the variable increases in size faster in the group with the larger regression coefficient, than in the other group (relative to SVL). In the following sections, all analyses were performed using SVL as the covariate. In general, the regressions for adult males and females can be considered as starting to diverge (or not) from the regression for juveniles. A more fine-grained analysis of changes in the oldest juveniles and the youngest sexually mature adults would facilitate a better understanding of the transition from juvenile to adult, but this analysis was not undertaken here.

For ease of reading, significance values are presented below only if not presented in accompanying Figures or Tables. Data are available at DRYAD (https://doi.org/10.5061/dryad.h18931zjm/).

## Results

### Intraspecific comparisons

#### Sexual dimorphism in body length

**Fig. 2:**
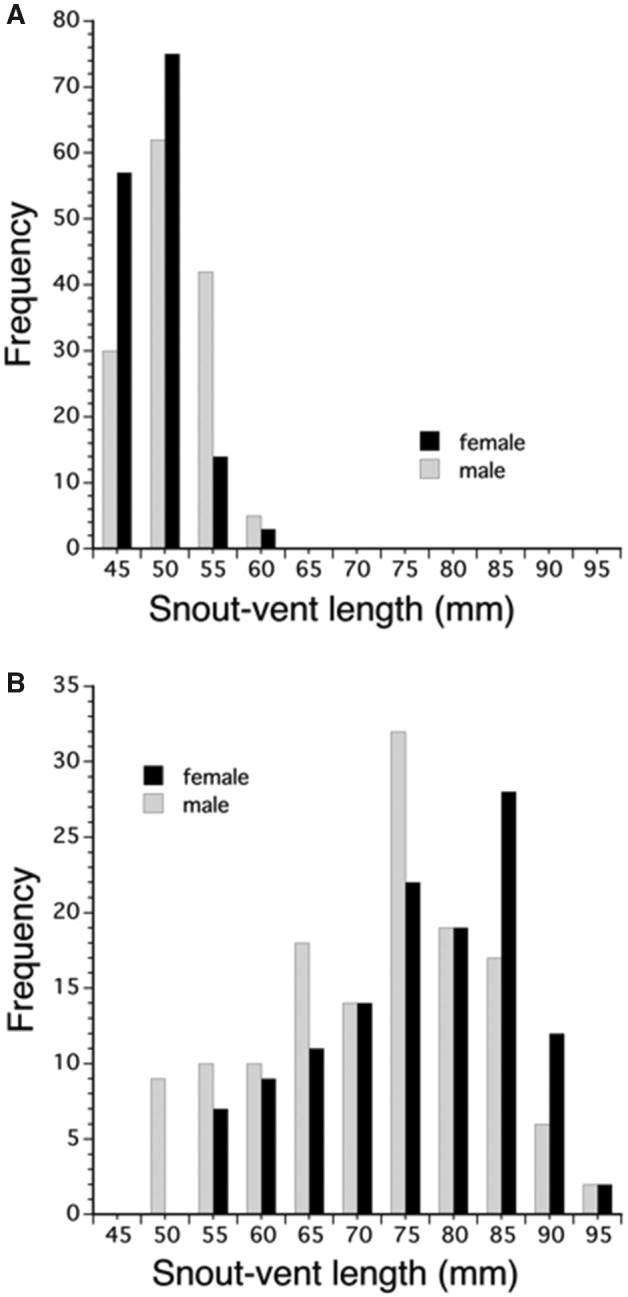
(**a**) Size class distribution for adult male (*n* = 139) and female (*n* = 149) *A. hardii*. There is a significant difference between the distribution of size classes for males and females, with more males in the larger size classes (Kolmogorov–Smirnov test, *P* < 0.01). *Aneides hardii* is the only species examined that shows male-biased sexual dimorphism in SVL. (**B**) Size class distribution for adult male (*n* = 137) and female (*n* = 124) *A. lugubris*. There is a significant difference between the distribution of size classes for adult males and females, with more females in the larger size classes (Kolmogorov–Smirnov test, *P* < 0.01). *Aneides lugubris* is the only species examined that shows female-biased sexual dimorphism in SVL.

Size (SVL) distributions were different between male and female *A. hardii* (Kolmogorov–Smirnov test, D = 0.225, *P* < 0.01); more males were in the larger size classes than females (Fig. 2A). For *A. aeneus* and *A. ferreus*, size (SVL) distributions were not different between adult males and females (*A. aeneus*: Kolmogorov–Smirnov test, D = 0.073, *P* = 0.91; *A. ferreus*: Kolmogorov–Smirnov test, D = 0.117, *P* = 0.34). Size (SVL) distributions were different between adult male and female *A. lugubris* (Kolmogorov–Smirnov test, D = 1.0, *P* < 0.01); more females were in the larger size classes than males (Fig. 2B). See Table 1 for SVL measurements.

**Table 1:** Range and mean for SVL, HW, HL, trunk length, and tail length for the species of *Aneides* examined

	*A. hardii*		*A. aeneus*		*A. flavipunctatus*		*A. ferreus*		*A. lugubris*	
	Males	Females	Males	Females	Males	Females	Males	Females	Males	Females
SVL range (mm)	45.2–62.2	45.2–61.9	42–71.9	43.2–67.0	54.4–86.0	54.5–87.0	51.9–76.4	50.3–80.0	51.1–98.7	55.0–99.3
Mean ± SD	52.9 ± 3.8	51.4 ± 3.4	52.9 ± 5.8	52.9 ± 5.7	68.5 ± 7.0	67.6 ± 7.2	63.0 ± 5.2	62.5 ± 5.2	74.2 ± 11.3	78.5 ± 10.1
*N*	139	149	201	84	103	94	113	153	137	124
HW range (mm)	6.2–10.1	6.1–8.0	6.6–13.6	6.8–11.3	8.0–15.3	7.0–13.1	7.5–13.4	7.0–12.8	8.1–20.0	8.7–18.7
Mean ± SD	7.8 ± 0.89	6.9 ± 0.41	9.0 ± 1.4	8.6 ± 1.1	10.5 ± 1.5	9.6 ± 1.2	10.4 ± 1.3	9.3 ± 1.0	13.5 ± 2.8	13.8 ± 2.1
*N*	139	149	201	84	103	94	113	153	137	123
HL range (mm)	10.2–14.7	10.1–13.1	10.2–17.5	10.7–16.6	12.6–20.3	12.7 ± 19.4	12.3–18.1	12.2–18.2	13.2–25.9	13.6–25.5
Mean ± SD	12.4 ± 0.92	11.5 ± 0.61	13.4 ± 1.5	13.1 ± 1.3	16.1 ± 1.5	15.3 ± 1.3	15.2 ± 1.3	14.7 ± 1.1	20.0 ± 3.1	20.7 ± 2.5
*N*	139	149	201	84	103	94	113	153	137	124
Trunk range (mm)	24.6–31.8	23.8–32.6	20.0–36.5	20.4–34.6	31.7–41.0	28.7–40.4	26.4–40.5	25.8–43.2	24.6–48.4	25.9–50.1
Mean ± SD	28.4 ± 1.7	27.6 ± 2.0	25.4 ± 3.5	26.1 ± 3.8	35.0 ± 2.8	35.0 ± 2.7	32.4 ± 2.8	32.7 ± 3.2	37.0 ± 5.4	38.9 ± 5.3
*N*	39	50	136	57	16	17	68	95	59	70
Tail range (mm)	32.6–55.2	28.8–48.1	5.0–85.0	6.3–78.9	27.7–67.0	27.5–71.4	7.0–61.0	22.7–60.4	16.6–80.1	5.0–80.1
Mean ± SD	43.8 ± 4.8	38.4 ± 3.5	50.5 ± 13.3	47.0 ± 15.1	51.3 ± 7.2	51.6 ± 7.0	42.3 ± 9.7	43.3 ± 6.4	57.0 ± 11.8	59.6 ± 11.3
*N*	139	149	197	83	103	94	113	153	136	124

#### Sexual dimorphism in HW and HL

**Fig. 3:**
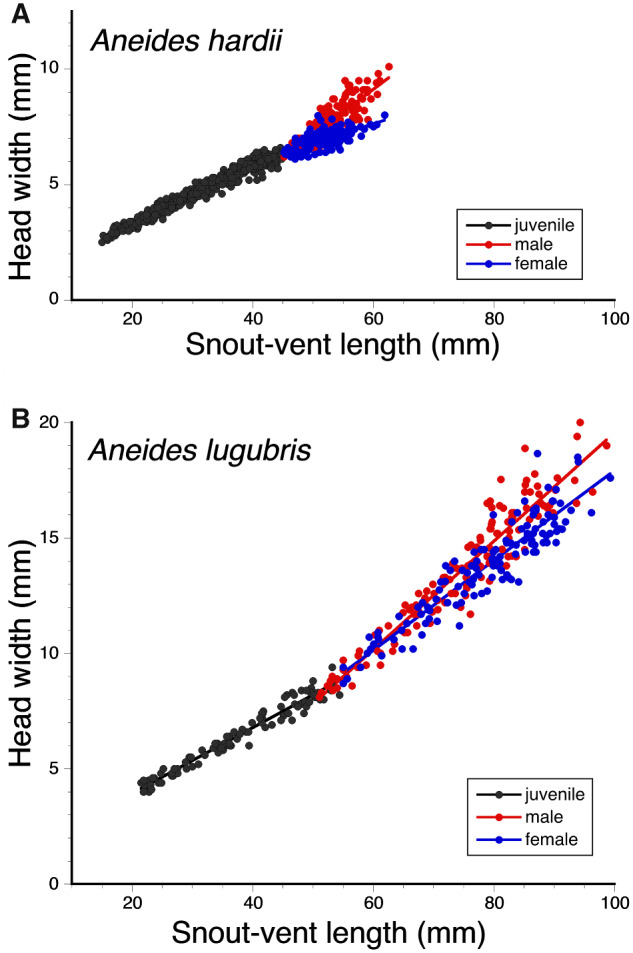
Regression showing the relationship between head-width and SVL for (**A**) adult male (*n* = 139), adult female (*n* = 149), and juvenile (*n* = 375) *A. hardii* and (**B**) adult male (*n* = 137), adult female (*n* = 123), and juvenile (*n* = 99) *A. lugubris*.

**Fig. 4:**
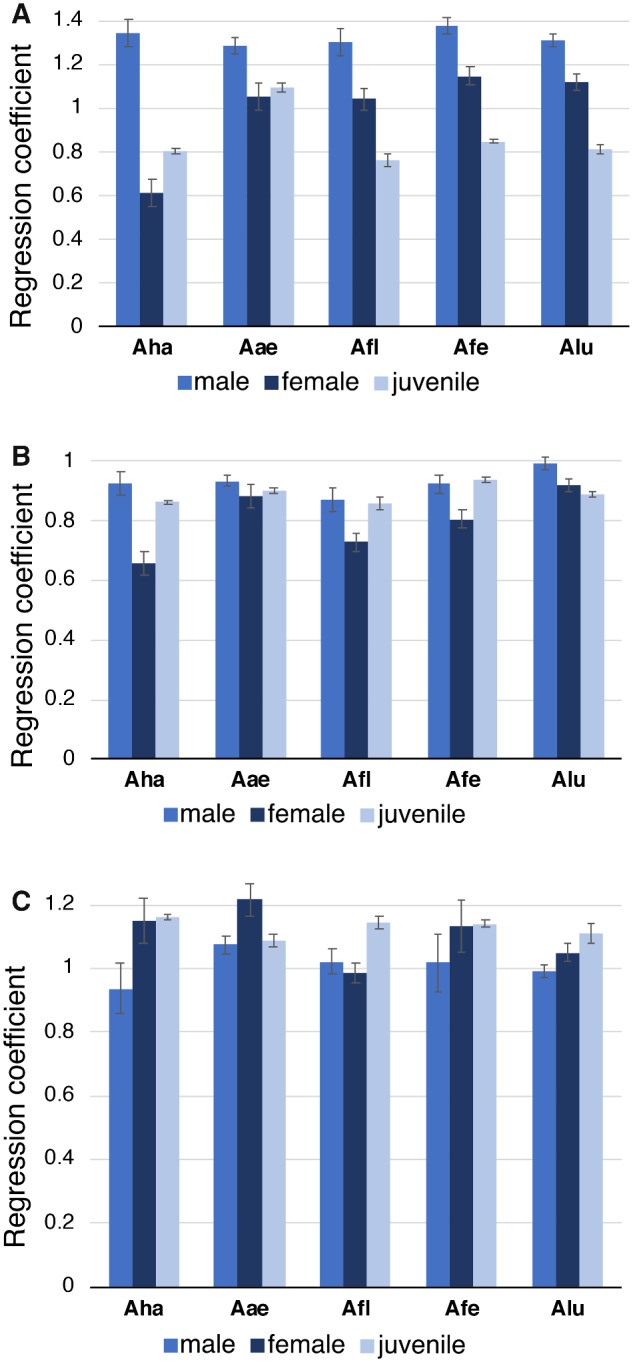
Bar graph of regression coefficients and standard errors for males, females, and juveniles for (**A**) HW, (**B**) HL, and (**C**) trunk (AG) length regressed against SVL, for the 5 species of *Aneides* examined. Note that male and female *A. hardii* have the greatest difference in regression coefficients for HW vs. SVL and the other species show reduced dimorphism. Aha: *A. hardii*; Aae: *A. aeneus*; Afl: *A. flavipunctatus*; Afe: *A. ferreus*; Alu: *A. lugubris*.

Comparisons between regressions from the ANCOVA for head-width and SVL (Fig. 3) show the degree of dimorphism for adult males and females in *A. hardii* and *A. lugubris*. Comparing the regression coefficients (for males, females, and juveniles) from the regression analyses provides an efficient measure of the degree of dimorphism for each of the morphological variables (Fig. 4). For HW, for example, the degree of dimorphism is greatest in *A. hardii* but all species show some dimorphism in the relationship between head-width and SVL with males having greater regression coefficients and wider heads relative to SVL, than females. *Aneides hardii* is the only species examined in which the regression coefficient for females was less than that for juveniles (Fig. 4A).

For *A. hardii, A. aeneus, A. ferreus*, and *A. lugubris*, there was a significant difference between regression coefficients for males, females, and juveniles (Tables 2 and 3; Fig. 4). From planned comparisons between adult males and females for all species, males had a relatively wider head (greater regression coefficient) than females relative to body length; this difference was greatest for *A. hardii* (Fig. 4A).

**Table 2:** Three-way comparison ANCOVA tests for equality of slopes between adult males, adult females, and juveniles for the species of *Aneides* examined

	HW		HL		Trunk length	
	*F*	*P-*value	*F*	*P-*value	*F*	*P-*value
*A. hardii*	51.61	<0.0001	12.52	<0.0001	3.59	0.029
*A. aeneus*	45.02	<0.0001	0.96	**0.38**	3.34	0.037
*A. flavipunctatus*	91.16	<0.0001	8.00	0.0004	2.64	**0.078**
*A. ferreus*	37.79	<0.0001	6.26	0.0021	4.61	0.011
*A. lugubris*	112.14	<0.0001	9.41	0.0001	3.99	0.021

See Fig. 4 for a graphical representation of these results (of regression coefficient values). Numbers in bold are nonsignificant comparisons

**Table 3:** Planned two-way comparison ANCOVA tests for equality of slopes between adult males and adult females

	HW		HL		Trunk length	
	*F*	*P-*value	*F*	*P-*value	*F*	*P-*value
*A. hardii*	66.15	0.0001	24.42	<0.0001	4.99	0.028
*A. aeneus*	10.2	0.002	1.46	**0.228**	7.16	0.0003
*A. flavipunctatus*	16.83	<0.0001	10.12	0.0017	0.12	**0.733**
*A. ferreus*	9.59	0.0022	5.71	0.017	5.59	0.019
*A. lugubris*	15.43	<0.0001	5.90	0.016	2.65	**0.106**

Those with nondifferent slopes were then tested for differences in intercepts. See Fig. 4 for a graphical representation of these results (of regression coefficient values). Numbers in bold are nonsignificant comparisons.

For *A. hardii, A. ferreus*, and *A. lugubris*, there was a significant difference between regression coefficients for males, females, and juveniles for HL using SVL as the covariate (Fig. 3; Tables 2 and 3). From planned comparisons between adult males and females, males had greater regression coefficients than females and relatively longer heads than females relative to body length (Fig. 3). Only for *A. aeneus*, there was no difference among males, females, and juveniles among regression coefficients for HL, but adult males had relatively longer heads than adult females (intercepts were different, *F* = 36.82, *P* < 0.0001).

#### Sexual dimorphism in trunk length

The relationship between trunk length and SVL was different for males, females, and juveniles for *A. hardii*, *A. aeneus*, *A. ferreus*, and *A. lugubris*. Planned comparisons between adult males and females showed that females had a larger regression coefficient for trunk length, and a relatively longer trunk, than males of *A. hardii*, *A. aeneus*, and *A. ferreus*. For *A. lugubris*, the regression coefficients for males and females were not different (*P* = 0.11), but the intercepts were with females having a larger trunk length relative to body length than males (intercepts were different, *F* = 16.16, *P* < 0.0001). There was no difference between regression coefficients for male, female, and juvenile *A. flavipunctatus* but adult females did have longer trunk lengths than males for a given body length (intercepts were different, *F* = 13.22, *P* = 0.001). For *A. hardii*, sexual dimorphism in trunk length was apparent in counts of vertebral elements as well; more females had 17 trunk vertebrae and fewer 16 trunk vertebrae than males did (*X^2^* [df = 1, *N* = 224] = 5.26, *P* = 0.02).

### Interspecific comparisons

**Fig. 5:**
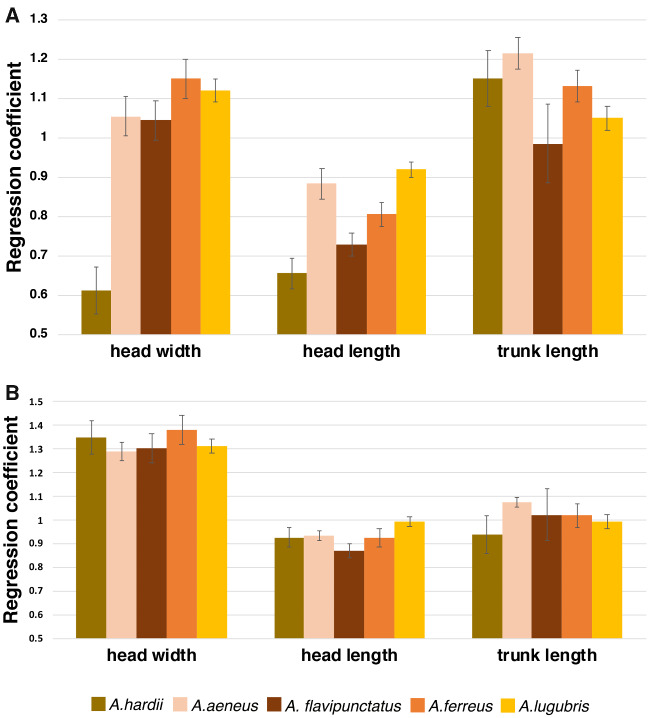
Interspecies comparisons of regression coefficients and standard errors within (**A**) female and (**B**) male *A. hardii*, *A. aeneus*, *A. flavipunctatus, A. ferreus*, and *A. lugubris* for HW, HL, and AG length regressed against SVL.

To compare the relationship between HW, HL, and AG length relative to SVL across species, comparisons were made separately for males and females using ANCOVA (Fig. 5). For these tests, the Bonferroni correction was used with the significance level of *P* = 0.0025.

#### Females

ANCOVA revealed a significant difference among regression coefficients across females of the 5 species examined for HW (*F* = 14.78, *P* < 0.0001). Pairwise comparisons show that the regression coefficient for female *A. hardii* for HW with SVL as the covariate, was significantly different from the regression coefficients for females of the other species examined (*F* = 14.78, *P* < 0.0001; Fig. 5A). There was no significant difference between the regression coefficients for *A. aeneus, A. flavipunctatus, A. ferreus*, and *A. lugubris* (*F* = 1.12, *P* = 0.339). Thus, the heads of females of these 4 species of *Aneides* scaled similarly with respect to body size. There were significant differences between intercepts (adjusted means) for these 4 species (*F* = 314.5, *P* < 0.0001): *A lugubris > A. aeneus > A. ferreus > A. flavipunctatus.*

ANCOVA revealed a significant difference among regression coefficients across females of the 5 species examined for HL (*F* = 10.72, *P* < 0.0001; with *A. aeneus* and *A. lugubris* having larger regression coefficients than the other species). *Aneides hardii* had the shortest head (and was not different from *A. flavipunctatus* and *A. ferreus*) and *A. lugubris* and *A. aeneus* (both not different from *A. ferreus*), had the longest, relative to SVL.

In contrast to HW and HL, regression coefficients for AG length were not different across females for all species examined (*F* = 4.06, *P* = 0.003 [Fig. 5A]). For these intercepts, only *A. hardii* and *A. flavipunctatus* were not different from each other (*P* > 0.28), with the rank order of adjusted means as follows: *A. hardii*, *A. flavipunctatus > A. ferreus > A. aeneus > A. lugubris*.

#### Males

In contrast to the pattern in females, in males there was not a significant difference among regression coefficients for HW (*F* = 0.44, *P* = 0.78; Fig. 5B), HL (*F* = 3.36, *P* = 0.0097), or AG length (*F* = 1.72, *P* = 0.14), among the 5 species examined. These variables scaled similarly to body length across these species. The intercepts for the HW regressions were all different (*P* < 0.001) except for *A. hardii* and *A. flavipunctatus* (*P* > 0.0025). The rank order of male head-width, adjusted for SVL, was *A. aeneus* > *A. ferreus* > *A. lugubris* > *A. hardii*, *A. flavipunctatus*. For HL, all intercepts were significantly different (*F* = 6870, *P* < 0.0001) with the rank order of adjusted means as follows: *A. lugubris > A. aeneus > A. ferreus > A. flavipunctatus > A. hardii*. There was a significant difference among intercepts for trunk lengths (*F* = 140.5, *P* < 0.0001), with *A. flavipunctatus* and *A. hardii* (*P* = 0.99) and *A. aeneus* and *A. lugubris* (*P* = 0.13) not different from each other. The rank order for trunk length was as follows *A. flavipunctatus*, *A hardii* > *A. ferreus* > *A. aeneus*, *A lugubris*.

## Discussion

Along with the outgroups, *Desmognathus* and *Phaeognathus*, *A. hardii* shows the putative ancestral pattern of sexual dimorphism in SVL, with males larger than females. Males are longer in SVL primarily because they have a longer head than females. Even though males are longer than females, female *A. hardii* have relatively longer trunks (AG length) than males. This pattern is observed in other plethodontids; female *Desmognathus quadramaculatus* and *Desmognathus aeneus* have significantly longer trunks than males do (Bakkegard and Rhea 2012). This pattern is also common in the “true” salamander subgroup of the Salamandridae (Malmgren and Thollesson 1999; Pogoda and Kupfer 2018).

In comparison with the outgroups, *Desmognathus* and *Phaeognathus*, *A. hardii* shows a derived pattern of sexual dimorphism in HW, with males having a wider head than females. This is consistent with male skulls being more heavily ossified than those of females and showing increased development of particular structures associated with strengthening of the skull, such as development of the otic crest and coronoid process, and extensive overlap of bony elements (Wake 1963). The otic crest and coronoid process serve as attachment sites of the adductor mandibulae muscles, which close the jaw. Relative to SVL, female heads scaled more similarly to heads of juveniles than to those of adult males.

The patterns of dimorphism in the other species of *Aneides* examined differ substantially from those of *A. hardii*. First, for all other species of *Aneides* examined, including *A. flavipunctatus* (Lynch 1981; Staub 2016), there is no male-biased size dimorphism; these other species show a derived pattern of sexual monomorphism in body length, with males and females showing no difference in size distributions or, interestingly, with females larger than males (in *A. lugubris*). These results are consistent with previous studies on *A. lugubris* (Lee et al. 2012). There may be population variation in size relationships, however; Waldron and Pauley (2007) noted that female *A. aeneus* were larger than males, and 2 of 12 populations of *A. flavipunctatus* showed size dimorphism (Lynch 1981).

A second way the other species of *Aneides* differ from *A. hardii* is in the degree of head dimorphism. Although sexual dimorphism in head size is present in all species of *Aneides*, the degree of dimorphism is dramatically less in species other than *A. hardii*, as seen in the difference in the regression coefficients between males and females (Fig. 4). This decrease in the degree of dimorphism in the other species is the result of adult females having hypertrophied jaw muscles, similar to adult males, rather than the ancestral state of no jaw muscle hypertrophy in either sex. While males of a few other plethodontid species have hypertrophied jaw muscles, it tends to be a species-specific characteristic (e.g., *Eurycea aquatica, Eurycea cirrigera* [Alcorn et al. 2013], *Eurycea wilderae* [Sever 1979; Pierson et al. 2019], and *Phaeognathus hubrichti* and *D. aeneus* [Bakkegard and Rhea 2012]). One similarity between the patterns shown in *A. hardii* to the other species of *Aneides* examined here is dimorphism in trunk length; females had longer trunks than males (Fig. 4 and Table 1).

Comparing males and females interspecifically shows the patterns of HW dimorphism in a different way. Because female *A. hardii* lack jaw muscle hypertrophy and females of other species have jaw muscle hypertrophy, the relationship between HW and SVL for female *A. hardii* is different from females of the other species examined (Fig. 5A). The regression coefficient for female *A. hardii* was significantly lower than the others, and thus relative to SVL, larger female *A. hardii* had narrower heads. HW scaled similarly to body length among females of the other 4 species examined. In contrast to females, the relationship between HW and SVL scaled similarly for males of all 5 species examined, including *A. hardii* (Fig. 5B).

Because of the difference in HW between male and female *A. hardii* and because of their male-biased size dimorphism, this species has the greatest degree of sexual dimorphism in the genus. While the other species of *Aneides* examined here show some dimorphism, it is slight because females have hypertrophied jaw muscles similar to those of males, and females are not shorter in body length than males. At sexual maturity of these other species, heads of both males and females begin to develop secondary sexual characteristics (hypertrophied jaw muscles) not seen in juveniles, as evidenced by larger regression coefficients for females compared to juveniles. An exception to this is with *A. aeneus*, in which the regression coefficient for juveniles appeared greater than the regression coefficients for juveniles of other species (Fig. 4A). The end result is that *A. aeneus*, *A. flavipunctatus*, *A. ferreus*, and *A. lugubris* show derived monomorphism in HW and SVL relative to outgroups *Desmognathus* and *Phaeognathus*, which show sexual dimorphism in SVL and the putatively ancestral monomorphism in HW.

How the dimorphism arises ontogenetically is due to a difference in relative HW and SVL growth (Staub 2016). As male and female *A. flavipunctatus* get larger, the rate of growth decreases for both body length and HW, but in males, the rate of HW growth does not slow down as much as it does in females (Staub 2016). The result is a relative burst of HW growth, relative to SVL, at sexual maturity in males (Staub 2016). Investigating the dynamics of growth rates at a finer scale and under more controlled conditions would help identify the proximate causes of the dimorphic and derived monomorphic patterns. Furthermore, comparing the growth patterns to species of *Desmognathus* would be informative as well, as this genus is characterized by large, powerful heads and jaws (though without dimorphism) relative to juveniles (Camp et al. 2019).

Mapping the patterns of morphological variation on the most recent phylogeny of *Aneides* reveals 2 hypotheses for the evolution of derived monomorphism in the genus (Fig. 1B and C). The first (Fig. 1B) has derived monomorphism evolving twice—once in the *A. aeneus* group and once in the *A. lugubris* group. In this case, the hypertrophy of male jaw musculature itself is a synapomorphy of the genus and subsequent hypertrophy of female jaw musculature and increase in SVL occurred independently in the *A. aeneus* and *A. lugubris* clades to result in derived monomorphism. This hypothesis is supported by earlier work that argued, based on an osteological study of *Aneides*, *Plethodon*, and *Ensatina*, that *A. hardii* is most similar to the most recent common ancestor of *Aneides* (Wake 1963). The second hypothesis (Fig. 1C) is that derived monomorphism evolved as a synapomorphy of *Aneides* with *A. hardii* females subsequently reverting to the ancestral state (shorter SVL and narrower heads). Because the number of characters used in this analysis is low and questions remain as to how to treat dimorphic traits on a phylogeny, these 2 hypotheses remain unresolved. Furthermore, these hypotheses are based on knowing the ancestral state of sexual dimorphism of (*Desmognathus* + *Phaeognathus*) + *Aneides* which needs further examination. Camp et al. (2019) suggest that the evolution of sexual dimorphism in *Desmognathus* was facilitated by sexual selection in males. A similar study analyzing types of selection in both males and females would be valuable to understand the evolution of derived monomorphism. Future work that includes the recently described species of *Aneides* (Patton et al. 2019; Reilly and Wake 2019) and examines how to treat sexually dimorphic and derived monomorphic traits on phylogenies would also be worthwhile.

Evaluating other lineages that show monomorphism, particularly when dimorphism is predicted, may reveal other cases of derived monomorphism. For example, female-biased size dimorphism was predicted for the Mediterranean salamanders *Salamandra atra* and *Mertensiella caucasica* (Reinhard et al. 2015). While there was sexual dimorphism in limb shape, unexpectedly there were no differences in size between males and females (Reinhard et al. 2015). Expanding the phylogenetic analysis of sexual monomorphism and dimorphism, as Pogoda and Kupfer (2018) have done for the Salamandridae, to include derived monomorphism and other salamander lineages would identify interesting evolutionary patterns, particularly when put in context of ecological and life history characteristics.

### Sexual dimorphism, derived monomorphism, and social selection

**Fig. 6:**
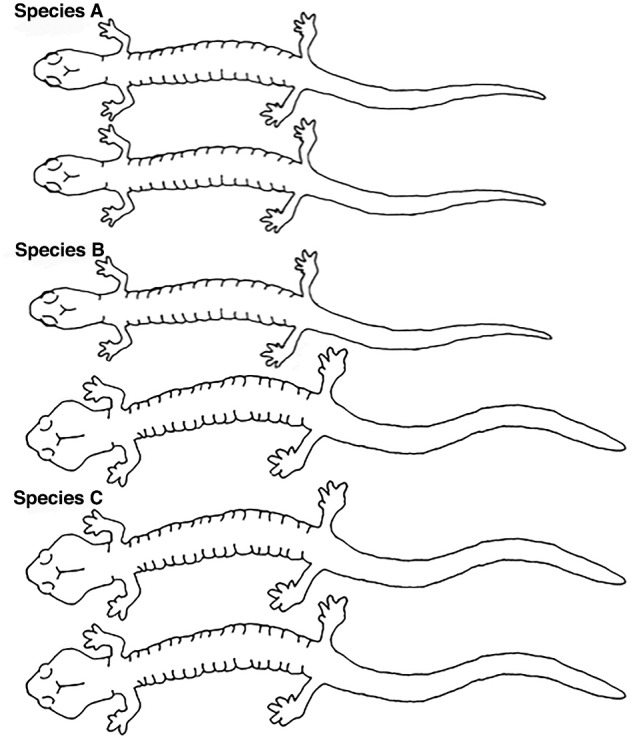
Schematic diagram of the proposed step-wise evolution of derived monomorphism (Species C) from an ancestrally monomorphic (Species A) state. Sexual dimorphism (Species B) can be a transitional stage between the two types of monomorphism.

The patterns of morphological variation in *Aneides* are consistent with the hypothesis that social selection has been important in the evolution of derived monomorphism in the genus (Fig. 6). As West-Eberhard (1983) predicted, there is character exaggeration in both sexes in 4 of the species examined. Unlike the pattern in *A. hardii*, there is no male-biased size dimorphism in the other species of *Aneides*. The absence of male-biased size dimorphism suggests that females have “caught up” with the level of morphological expression found in males in body length and head morphology. *Aneides lugubris* is especially interesting in this case, because females have not only “caught-up” with males but have attained the more typical size relationship in amphibians, in which females are larger than males.

*Aneides hardii*, *A. flavipunctatus*, and *A. lugubris* display agonistic behavior and *A. hardii*, *A. aeneus*, *A. flavipunctatus*, *A. ferreus*, and *A. lugubris* all show the diagnostic semi-circular upper-jaw-shaped scars from it (Staub 1993). Both males and females had these scars, although male *A. flavipunctatus* and *A. ferreus* had significantly more scarring than females. Interestingly, in *A. lugubris*, the amount of scarring was not different between the sexes (Staub 1993). Agonistic interactions are typically associated with male-biased sexual size dimorphism (Shine 1979), although in *Desomgnathus* this pattern does not hold (Halliday and Verrell 1986). The lack of male-biased size dimorphism in 4 of the 5 species of *Aneides* examined suggests that females are also under selective pressure to become larger and have stronger jaws. Having hypertrophied jaw muscles that are advantageous in contests with conspecifics may also enable eating larger and harder-bodied prey, for example. At the extreme end of the morphological variation within *Aneides* is *A. lugubris*, which exhibits peramorphosis of traits (more extreme development than in the ancestral condition) in both females and males (Wake et al. 1983). These traits are associated with strengthening the skull, such as monocuspid teeth throughout life and co-ossification of the skull and skin (Wake et al. 1983). More focused studies on derived monomorphism and the underlying agents of social selection in this group would be valuable to understanding its evolution. Furthermore, studying the genetic variation within and between populations and species as it relates to head shape, as Adams (2011) has done with species of *Plethodon*, would add another component to understanding variation within and between species. The “true” salamanders in the Salamandridae clade also show interesting patterns of size monomorphism between females and males (Reinhard et al. 2015; Pogoda and Kupfer 2018); a more comprehensive study of monomorphism, dimorphism, and derived monomorphism across salamander lineages may well reveal some previously undiscovered evolutionary patterns.

Social selection (including sexual selection) is obvious when it acts counter to natural selection, producing traits that are maladaptive to survival (see Kirkpatrick 1987, for a review). In this case, sexual dimorphism can be slow to evolve (Lande 1980). Synergism, however, between natural and social selection can explain the evolution of derived monomorphism from a sexually dimorphic state (see West-Eberhard 2003). Price and Birch (1996) argue that the rate of evolution from dimorphism to derived monomorphism is actually greater than the rate of monomorphism to dimorphism, at least in passerine birds.

What proximate mechanism could drive the expression of derived monomorphism? In *Anolis* lizards, the administration of testosterone to juvenile females decreased the expression of female-biased genes and increased the expression of male-biased genes, resulting in less sexual dimorphism (Cox et al. 2017). Because androgens play a normal role in female development (see Staub and De Beer 1997 for a review), a slight quantitative change in circulating androgen levels or in tissue sensitivity could account for the expression of derived monomorphic traits in females. This pattern has been observed in the frog *Pelophylax esculentus*. In this species, plasma levels of androgens are high in females, and the skin (with its associated glands) is considered a secondary sexual characteristic because of its high level of androgen receptors (Delrio et al. 1979). In another example, high levels of androgens produced by ovotestes in female Iberian moles result in several male-to-female derived monomorphic traits (Real et al. 2020). More studies investigating the normal role of androgens in females and other relevant hormones would help us understand their normal role in the development of derived phenotypes in females and males.

### Summary

As West-Eberhard (1983) predicted from social selection, there is character exaggeration within *Aneides*: females express derived monomorphic traits in HW and SVL. Because monomorphism has not been studied as intensely as sexual dimorphism, few studies have explored the selective forces underlying the evolution of derived monomorphism. Studies investigating the specific types of selection on females and males would be valuable to help understand the evolution of derived monomorphism in this group. Future work that integrates the underlying genetic architecture, gene expression, or sex-specific selective regimes for the evolution of derived monomorphism, such as Connallon (2015) has done for the analysis of sexual dimorphism and Pointer et al. (2013) have done for the differences in gene expression underlying variation in dimorphic traits, would be a significant contribution to understanding the evolution of derived monomorphism. A closer examination of the relationship between juvenile and adult growth rates would help determine when ontogenetic shift occurs for males and females.

Additionally, the occurrence of derived monomorphic features in females may well be more common than traditionally believed. For example, females of 2 species of toads (genus *Melanophryniscus*) have nuptial-like pads which typically are only found in males (Jeckel et al. 2019). Including females in analyses, especially for traits that are thought to be only present in males, is important to document these patterns of derived monomorphism.

The initial evolution of sexual dimorphism sets the stage for the evolution of derived monomorphism via social selection (Fig. 6). Regardless of the specific origin of the dimorphism, characters present in one sex can be selected in the other sex, as long as the original difference between the sexes is itself not maintained by stronger selective pressure. The patterns of SVL and HW dimorphism described for *Aneides* support the hypothesis that sexual dimorphism can be a transitional stage in the evolution of new species-specific morphologies.

## Supplementary Material

obaa044_Supplementary_Data
