# Supplementary material for: The Evolution of Derived Monomorphism From Sexual Dimorphism: A Case Study on Salamanders
Source: Integr Org Biol. 2020 Dec 21;3(1):obaa044. doi: 10.1093/iob/obaa044 (PMC7885154; doi:10.1093/iob/obaa044)
Supplement: obaa044_Supplementary_Data [file obaa044_supplementary_data.docx]

Appendix 1. Specimens examined.

(NLS = personal collection of Nancy Staub, to be accessioned by the Museum of Vertebrate Zoology)

*A. aeneus:*

American Museum of Natural History: 25583-4, 25586-9, 25591-3, 25595, 25597-604, 36123-29, 37190-1, 37584-9, 38007, 40136-7,41624-9, 41637-42, 41790, 44963, 45117-20, 45218, 50162-4, 124703-31

Field Museum of Natural History: 193865-882, 193884-903

University of Michigan Museum of Zoology: 59048, 63078, 74545, 75506 (2), 77394, 85557 (15), 86583, 88588 (7), 95742, 95743 (3), 95942 (5), 95943, 127945 (2), 127948 (2), 128643 (3), 128644 (8), 128645(3), 128651, 109107 (3), 109174, 125578, 128771 (4), 130675 (31), 136379, 136586, 136588-89, 137483 (2), 138648 (2), 109108 (10), 109105 (19), 109106 (18), 109686, 110494 (2), 111396 (5), 125661, 173024

Museum of Vertebrate Zoology: 15843, 32571, 52959-70, 92086-92117, 175795-97, 178574-85

NS: 453-487, 999(12)

*A. ferreus*

Museum of Vertebrate Zoology: 192976, 192984, 192994, 201438, 201441, 201447, 201456, 201467, 201469, 201471, 201473, 201474, 201531, 201533, 201534, 201538, 201540, 201561, 201567-9, 201576-8, 201634-5, 201662, 201664-5, 201667-8, 201673, 201690, 201699, 201702, 201704, 201706, 201716, 201718-9, 201721-3, 201724-5, 201739-45, 201875, 201877, 201895, 201899-901, 201903, 201905-7, 201933, 201936-9, 201942, 201980, 201982-5, 201987, 201989, 201992, 201994-7

Joseph Beatty catalogue numbers: 41, 49, 59, 61, 88, 130, 136, 178-80, 235, 238, 311, 313, 332, 337, 518, 535, 564, 648, 1001, 1113-6, 1008-13, 1016-18, 1020, 1022-3, 1026-7, 1029-30, 1033, 1037-8, 1040

*A. flavipunctatus*

Museum of Vertebrate Zoology: 90365-70, 90373-81, 90383-87, 90393-95, 99463-4, 158442-3, 187712, 193025, 193057

*A. hardii*

R. Highton specimens from collections 75-180, 76-136, 77-122

NLS: 36-149, 500-975, 984-1024

for osteological analyses: NLS: 713, 719, 725, 728, 735, 743, 746, 749, 753, 758, 761, 767, 770, 773, 792, 795-6, 816, 821-2, 824, 833, 835, 840, 851-4, 861, 872, 874, 886, 889, 895, 898, 912, 915, 917, 919, 922, 927, 932, 944, 956, 970, 973, 975

*A. lugubris*

Museum of Vertebrate Zoology: 5673, 5676, 5678, 5681-2, 5685, 7366, 7373, 50239, 50251, 50252, 50254, 55907-9, 55911, 55915-9, 55990, 57355-60, 58228-34, 59708-13, 60121-24, 60906, 62263, 62426, 62535, 62537-40, 66260, 76947-56, 76958-82, 78164, 79167, 79170, 79541, 90299-359, 100125-6, 107277, 117617-8, 117629-30, 124654-778, 124819-20, 124842-4, 124846, 137152-3, 145722-34, 147587, 147665-6, 157970-1, 157973-77, 157979, 157982-3, 158401-4, 158406-11, 158439-41, 172613, 172615-7, 173065, 173080-6, 173094, 173101-3, 177881, 178612, 178617, 181450-1, 181929, 187338-42, 187714, 193541
